# Trans-saccadic priming in hemianopia: Sighted-field sensitivity is boosted by a blind-field prime

**DOI:** 10.1016/j.neuropsychologia.2012.02.006

**Published:** 2012-04

**Authors:** Kay L. Ritchie, Amelia R. Hunt, Arash Sahraie

**Affiliations:** Vision and Attention Laboratories, School of Psychology, University of Aberdeen, Aberdeen AB24 3FX, UK

**Keywords:** Remapping, Hemianopia, Eye movements, Attention

## Abstract

We experience visual stability despite shifts of the visual array across the retina produced by eye movements. A process known as remapping is thought to keep track of the spatial locations of objects as they move on the retina. We explored remapping in damaged visual cortex by presenting a stimulus in the blind field of two patients with hemianopia. When they executed a saccadic eye movement that would bring the stimulated location into the sighted field, reported awareness of the stimulus increased, even though the stimulus was removed before the saccade began and so never actually fell in the sighted field. Moreover, when a location was primed by a blind-field stimulus and then brought into the sighted field by a saccade, detection sensitivity for near-threshold targets appearing at this location increased dramatically. The results demonstrate that brain areas supporting conscious vision are not necessary for remapping, and suggest visual stability is maintained for salient objects even when they are not consciously perceived.

## Highlights

► Awareness of a blind-field stimulus increased when a saccade was about to bring that stimulus into the sighted field. ► A blind-field prime facilitated detection of a near-threshold sighted field stimulus in the same location. ► Post-saccadic enhanced sensitivity in the sighted field was specific to the spatial location previously occupied by the blind-field target.

## 1 Introduction

### 1.1 Visual stability across saccades

When the eyes move, the image on the retina shifts, and in visual cortex, the representation of a stimulus in a given spatial location shifts from one population of neurons to another. Eye movements ought to produce the sensation of an unstable visual world, but despite making multiple saccadic eye movements each second, perception is subjectively stable and continuous. One explanation for perceived visual stability across eye movements is that the visual system is able to predict the consequences of saccadic eye movements before they begin through a process known as saccadic remapping.

Prediction seems to be important for the control and perception of one's own action. Efference copy (von Holst & Mittelstaedt, 1954), also referred to as corollary discharge (Sperry, 1950) is the idea that when the brain commands muscles to make a movement, a copy of this command is sent to sensory networks to provide information about the perceptual consequences of the movement. In the case of saccades, efference copy has been argued to play a role in monitoring the location of objects in visual space by providing a signal that leads to the transfer of activity on retinotopic maps to the location on the retina where those objects will appear after an eye movement (for a review, see Wurtz, 2008). This transfer is known as saccadic remapping, and was first observed in a now-classic experiment (Duhamel, Colby, & Goldberg, 1992) in which visual cells in the lateral intraparietal area (LIP) increased firing around the time of an eye movement for stimuli that were expected to be shifted into their receptive fields by an eye movement, but had not yet arrived. Similar responses have been found in the frontal eye fields (FEF) (Umeno & Goldberg, 1997, 2001) and extrastriate areas (Nakamura & Colby, 2002). This remapping response was initially characterized as classic receptive fields briefly shifting to take in information from a different location in space just before an eye movement (Duhamel et al., 1992; Sommer & Wurtz, 2003; Zirnsak, Lappe, & Hamker 2010). It has recently been suggested that the remapping process may be better described as an updating of attention pointers (Cavanagh, Hunt, Afraz, & Rolfs, 2010). That is, peaks of activity on retinotopic maps that keep track of attended locations and potential saccade targets are re-aligned to correspond with their expected post-saccade coordinates just before the eye movement. This re-alignment allows attention to be maintained at appropriate spatial locations when the eye movement shifts them across the retina.

At the single-unit level, in areas like LIP that code attended locations, it has not yet been possible to distinguish whether the remapping activity represents a shift of receptive fields or a transfer of activity from one population of cells to another. Research in human vision has supported the notion that attention plays a critical role in maintaining spatial representations across eye movements. For instance, attention boosts detection of location changes that occur during eye movements (Bridgeman, 1981). More recently it has been shown that when an eye movement sequence is planned, attentional benefits can be observed at the future retinotopic location of saccade targets in the sequence (Rolfs, Jonikaitis, Deubel & Cavanagh, 2010), and targets can be masked by noisy patterns when they appear in their future retinotopic location just before a saccade (Hunt & Cavanagh, 2011). This suggests that when attention is maintained on a peripheral target at the same time as a saccade is planned, attention begins shifting to the expected retinotopic coordinates of that target before the eyes move.

### 1.2 Hemianopia and blindsight

Early visual areas have been implicated in the remapping process, but to a lesser extent than LIP and FEF (Merriam, Genovese, & Colby, 2007; Nakamura & Colby, 2002). In other words, at the time of saccade onset most neurons in LIP respond to the predicted future location of attended stimuli, while most neurons in V1 respond veridically to the current location of stimuli. After the saccade has been completed, there may sometimes be a mismatch between the predicted location of attended objects in LIP and actual stimulation from those objects in V1, and this could provide the basis for detecting errors in saccade execution, or changes in the visual array that occurred during an eye movement. Therefore, early visual areas may play an important role in perceptual continuity by enabling comparison of the predicted locations of objects of interest to a veridical representation of retinotopic input after the saccade.

V1 lesions leave patients without phenomenal sight in the corresponding areas of their visual field, yet a range of residual visual abilities have been found in the blind fields, known collectively as blindsight (Weiskrantz, 1986; Weiskrantz, Warrington, Sanders, & Marshall, 1974). Perimietrically blind areas are defined as those in which there are zero decibels of sensitivity recorded during perimetry. In the perimetry procedure, participants are asked to press a button whenever they consciously perceive a small point of light. Despite being identified as “blind” according to this procedure, participants may show blindsight in perimetrically blind areas in response to a range of stimuli. Blindsight highlights the distinction between perception and awareness (Koch & Tsuchiya, 2007), as participants are often able to detect and discriminate stimuli presented in the blind field (Kentridge, Heywood, & Weiskrantz, 1999) without those stimuli entering awareness. The visual pathway from the retina to the superior colliculus has been implicated in supporting residual visual abilities (Leh, Ptito, Schönwiesner, Chakravarty, & Mullen, 2009; Rafal, Smith, Krantz, Cohen & Brennan, 1992; Sahraie et al., 1997), and the superior colliculus has been implicated in the remapping process by way of signals to FEF (Sommer & Wurtz, 2003). Representations of the “blind” stimulus that underlie above-chance detection could provide the basis for a signal that could be remapped to the sighted field before an eye movement. Blindsight therefore provides a unique opportunity to evaluate the role of early visual areas in remapping.

## 2 Experiment 1. Remapping of a stimulus that is removed before saccade onset

We first examined whether a blind-field stimulus that is detected above chance but not consciously perceived would be remapped before an eye movement. If a stimulus is remapped from the blind to the sighted visual field, we would expect enhancement in sensitivity for that stimulus, whether through detection or awareness.

### 2.1 Methods

#### 2.1.1 Participants

**Fig. 1:**
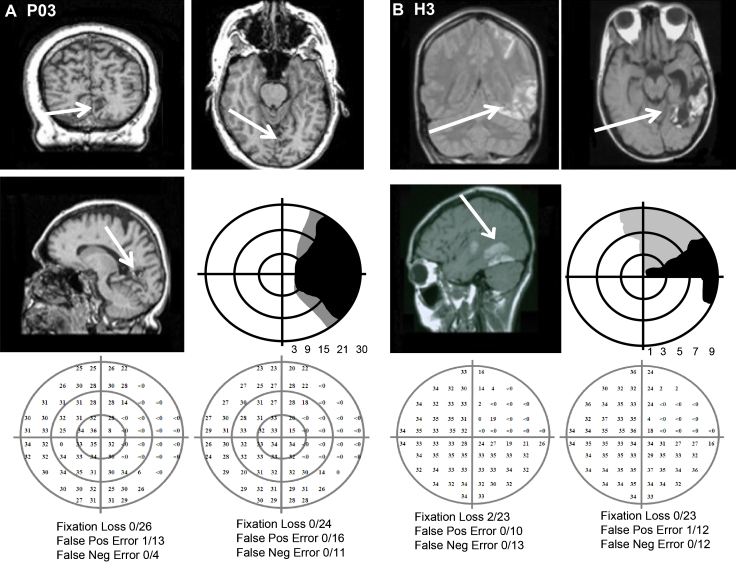
T1-weighted structural MRI scans, schematic representations of binocular visual fields (30-2), and individual central monocular fields (P03: 30-2, H3: 10-2) obtained using Humphrey Automated Visual Field Analyser for both patients. The test parameters indicate steady fixation. (A) P03; (B) H3.

Two participants volunteered for the study. P03 is a 62-year-old male who suffered a stroke 11 years prior to testing leading to a right homonymous hemianopia. He has no cognitive or motor impairments. The ischemic attack damaged the anterior medial aspect of the left occipital lobe and underlying white matter. P03 has previously participated in experiments (also referred to as P03 in Sahraie et al., 2003; and in Sahraie, Trevethan, & Macleod, 2008) and has shown above chance detection of 10° targets, with correct detection peaking at a spatial frequency of 1c/° (Sahraie et al., 2003) and a temporal frequency of 10 Hz (Sahraie et al., 2008). P03's visual fields were measured on 21st April 2010 using a standard automated perimetry (30-2 program, Humphrey Visual Field Analyser) as well as a binocular measure of sensitivity (Esterman binocular functional test) (Fig. 1A).

H3 is a 26-year-old female who was tested 5 years after suffering a left temporo-parietal haemorrhagic infarction. The damage resulted in a scotoma in the upper right quadrant of the visual field. H3 previously participated in a daily training study and showed increased sensitivity to low spatial frequency stimuli following a period of training (also referred to as H3 in Sahraie et al., 2010). Her visual fields were measured on 15th January 2008 using a standard automated perimetry (10-2 program, Humphrey Visual Field Analyser) as well as a binocular measure of sensitivity (Esterman binocular functional test) (Fig. 1B).

Testing took place over a number of sessions with each participant. Each session lasted no longer than 3 h, with frequent breaks. The experiments were conducted in order of 1–3, with each testing session containing only one experiment but multiple randomly interleaved blocks of each condition of that experiment. All testing was completed with P03 between May and July of 2010, and with H3 between June and October of 2011. Stimuli contrasts, durations, and locations were selected individually for each participant based on their relative sensitivity, and the size and shape of their blind field.

The study was granted full ethical agreement from the School of Psychology Ethics committee, and from the National Research Ethics Service, and informed consent was obtained from both participants prior to testing.

### 2.2 Materials

The blind-field stimulus was a circular sinusoidal vertical grating of one cycle-per-degree, at 90% contrast, subtending 10° for P03 and 3° for H3 at a viewing distance of 370 mm. Each participant's blind-field stimulus was also used as their sighted-field stimulus in this experiment. The stimuli were presented against a uniform grey background (37 cd/m^2^) on a ViewSonic Graphics Series G90fB 360 mm × 275 mm CRT monitor with a refresh rate of 85 Hz. A chin and forehead rest stabilised head position, and eye position was monitored using a desk-mounted EyeLink1000 system (SR Research Systems, Ontario, Canada) which uses infrared light to track the eye position, sampling the gaze direction at a rate of 1000 Hz.

#### 2.2.1 Procedure

**Fig. 2:**
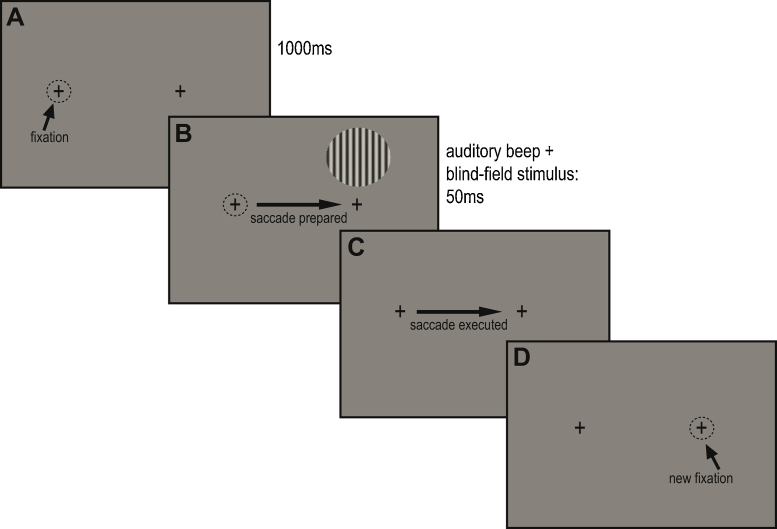
The sequence of events on a typical right-saccade trial in Experiment 1 for P03. (A) P03 fixates the left crosshair. (B) After 1000 ms, an auditory beep instructs him to shift his eyes to the right crosshair. A 10° grating is shown for 50 ms. (C) The saccade is executed, usually after the grating has disappeared (trials with saccadic latencies shorter than 50 ms were excluded). (D) P03 is fixating the right fixation point. Had the grating remained, it would now fall largely in his sighted field.

Fig. 2 illustrates a typical trial for P03. At the beginning of each trial, the participants were asked to fixate a black cross subtending 0.7°, on the left of the centre of the screen. This was followed by an auditory beep in all conditions. For conditions in which a saccade was to be made, the auditory beep signalled the participants to saccade to a second fixation cross. In the *blind* – *saccade right* condition, the second fixation cross was positioned to the right of the first (P03 (+20°, 0°), H3 (+15°, −6°)). For P03, we also included a *blind* – *saccade up* condition, in which the second cross was positioned 10° above the first. This condition was omitted for H3, whose blind-field size and shape rendered it impossible. In the *blind* – *no saccade* condition, the auditory beep was presented but there was no second fixation cross and no saccade was made. On half of all trials, a grating appeared for 50 ms, simultaneous with the offset of the beep. The short duration of the stimulus meant that in the conditions in which a saccade was to be made, the stimulus had offset before the saccade was initiated, and thus never appeared in the predicted post-saccadic location. In the blind-field conditions, the grating was presented in each participant's blind right visual field (first fixation cross to near edge of stimulus: (P03 (+15°, −9°), H3 (+13.5°, +1.5°)). In the *sighted field* condition, the stimulus was positioned in the location which would have been the post-saccadic location in the *blind* – *saccade right* condition, had the stimulus remained on the screen. For P03 there was a total of 90 trials in each condition with no trials excluded. For H3 there were 50 sighted-field trials, 227 trials (after 13 exclusions) in the *blind* – *no saccade* condition, and 194 trials (after 16 exclusions) in the *blind* – *saccade right* condition. (Trial exclusion criteria are described below.) Participants were informed that the target was present on half of the trials. Following the commentary key paradigm reported previously (Weiskrantz, 1998), the participants were asked to provide two responses at the end of each trial; the first indicating detection, and the second awareness. The detection response was a ‘yes’ or ‘no’ response, and if the participant was not sure, they were asked to guess. The participants would only give an awareness response on trials where their detection response had been positive. Both detection and awareness are measures of sensitivity. Responses were given verbally and recorded by the experimenter by pressing the corresponding buttons on a response box.

### 2.3 Data analysis

In all of the experiments, saccades were made from the left fixation cross. The EyeLink1000 system's drift correct feature ensured correct eye position at the start of each trial. Trials were excluded from analysis if eye movement errors brought the stimulus within 1° of the edge of the perimetrically defined blind field at the time it offset, or if a saccade was made in the no saccade trials, or not made in the saccade trials. No trials were excluded from P03's data. For H3, 7.6% of saccade trials and 5.4% of no-saccade trials were excluded. *d*-prime was calculated to measure sensitivity while taking response bias into account (Macmillan & Creelman, 1991). Bootstrapping was used to estimate 95% confidence intervals, that is, the sampling distribution of means for each condition was estimated by randomly sampling with replacement from the data set 10,000 times. Inferential statistics were based on the 95% confidence interval around the mean derived from the bootstrapping procedure. A *d*-prime value with confidence intervals which include 0 indicates chance performance. Performance between two conditions would be considered to be significantly different at the *α* = 0.05 level if the bootstrapped confidence interval around each mean does not overlap with the mean of the other condition.

### 2.4 Results and discussion

**P03**. Firstly we established that P03 was able to accurately target saccades to the second fixation cross. In the key condition (*blind - saccade right*), the cross to which P03 was required to saccade was located in his blind field. At the start of each testing session we showed P03 where the cross would be positioned and asked him to make a practice saccade from the first fixation cross to that location on the screen. In the key condition P03's mean landing position undershot on average by −1.68° (SD = 0.44°).

**Fig. 3:**
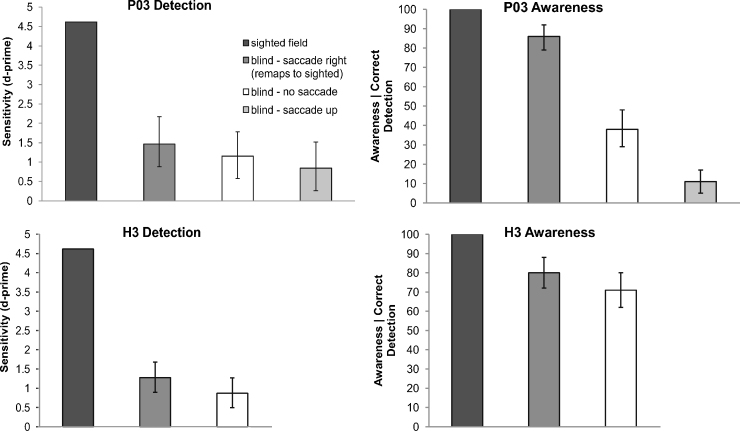
Data for P03 and H3 for Experiment 1. Left panels, sensitivity (*d*-prime) for targets shown in sighted field (dark), and for targets in the blind field before a rightward saccade (dark grey), which would remap the target into sighted field. The white and light grey bars are blind-field control conditions (*blind* – *saccade up* P03 only). P03 shows no significant difference between the three blind-field conditions, whereas H3 shows an increase in detection in the remapping condition. Right panels, the percent of trials on which participants reported being aware of the target out of the total number of trials on which the target was correctly detected. Awareness is elevated in both participants relative to the control conditions. Error bars represent the 95% confidence interval based on bootstrap resampling.

Results for P03 are shown in Fig. 3 (upper panels). Detection performance in the sighted field was 100%. Detection of the same grating in the blind field was 70% correct (*d*′ = 1.15). The introduction of a saccade which would have brought the stimulus into the sighted visual field did not significantly increase detection relative to the control conditions (see Fig. 3): *blind* – *no saccade*: *d*′ = 1.15, *saccade-up*: *d*′ = 0.85, *saccade-right*: *d*′ = 1.46, *p* > .05; *p*-values in this and all subsequent contrasts are based on bootstrapped confidence intervals (see Section 2.3). P03 reported that on trials for which he said he was aware of the stimulus he “…couldn’t see anything but just felt like something was there”. When asked, P03 said he could not describe the stimulus or even guess its shape. The rightward saccade did significantly increase awareness of the stimulus relative to conditions in which the stimulus remained in the blind visual field (*blind* – *no saccade:* 38%; *blind* – *saccade up*: 11%; *blind* – *saccade right*: 86%, *p* < .05). This increase in reported awareness in the key condition remains significant when response bias is controlled using a *d*-prime measure of awareness (*blind* – *no saccade*: *d*′ = 0.51; *blind* – *saccade up*: *d*′ = 0.31; *blind* – *saccade right*: *d*′ = 1.86, *p* < .05).

**H3**. As with P03, H3 was able to accurately target saccades to the second fixation cross despite it falling within her blind field. In the key condition (*blind - saccade right*), H3's mean landing position was −2.66° (SD = 0.58°) from the target.

Results for H3 are shown in Fig. 3 (lower panels). Detection performance in the sighted field was 100%. Detection of the same grating in the blind field was 70% correct (*d*′ = 0.87). The introduction of a saccade which would have brought the stimulus into the sighted visual field significantly increased detection relative to the control condition (*blind - no saccade: d*′ = 0.87, *blind* – *saccade right*: *d*′ = 1.27, *p* < .05). The rightward saccade also significantly increased awareness of the stimulus relative to the control condition (*blind* – *no saccade*: 71%; *blind saccade right*: 80%, *p* < .05). This increase in reported awareness in the key condition remains significant when response bias is controlled using a *d*-prime measure of awareness (*blind* – *no saccade*: *d*′ = 0.36; *blind* – *saccade right*: *d*′ = 1.28, *p* < .05). When asked about her awareness reports, H3 stated, “When I say ‘no’ there's nothing there. When I say ‘yes’ I’m pretty sure there's something there so I usually say ‘aware’, but I couldn’t say what”. As with P03, when asked to describe the blind-field stimulus, H3 could not.

This experiment demonstrated that both detection and awareness of a blind-field stimulus increase when it is about to be shifted into the sighted field relative to conditions with no saccade, and also relative to conditions with a saccade that would have shifted the stimulus from one part of the blind field to another one. We speculate that the significant increase in awareness occurred because a signal from the blind field that was just below the threshold for awareness was sometimes elevated above this threshold when it was remapped to the sighted field. H3 reported high awareness of the stimulus in her blind field without making a saccade, nevertheless the introduction of the saccade boosted both detection and awareness.

Our interpretation of the results is that the onset of the blind-field stimulus, though below the threshold for awareness, nonetheless attracted attention. When this attended location was just about to be shifted into the sighted field by a saccade, the predictive transfer of the associated activity into a sighted-field location boosted awareness. It is interesting to note that transferring activation to the intact hemisphere is not equivalent to that of presenting an actual stimulus in the sighted field, given that the same stimulus shown in the sighted field led to perfect detection and awareness (see Fig. 3). Based on these results, we suggest that remapping is only the first step in maintaining a representation across a saccade. That is, remapping shifts attention to the expected new location of a target of interest, but without a stimulus subsequently appearing in this retinotopic location as the saccade is completed, the benefits of the remapping effect cannot be measured. To test this idea, in the next experiment we examined the effect of a pre-saccadic blind-field stimulus on subsequent detection of a sighted-field stimulus presented in the same location.

## 3 Experiment 2. Effect of a pre-saccadic blind-field stimulus on detection of a near-threshold sighted-field stimulus in the same location

We presented the same supra-threshold blind-field stimulus for each participant as in Experiment 1 before the eye movement, and replaced it with a very dim (1% contrast for P03, 2% contrast for H3) version of the same stimulus just before the eye movement shifted the target into the sighted field. We compared detection of the dim target when preceded by a blind-field prime to detection when the same dim target appeared alone. Based on the results of Experiment 1, and should our interpretation be correct, we expected detection of the dim sighted-field target after the saccade to benefit from the prediction that a stimulus will be present in that location after the saccade. Therefore we expected that sensitivity would increase significantly when both the blind- and sighted-field stimuli were presented.

### 3.1 Procedure

In this experiment, eye movements were recorded at a rate of 2000 Hz. The task was to indicate whether or not the stimulus had been presented during each trial. Participants fixated a cross on the left side of the screen for 1000 ms, after which an auditory beep sounded. The sound was the cue to saccade to a second cross positioned to the right of the first fixation: P03 (+20°, 0°), H3 (+15°, −6°). Stimuli were presented in the same locations for each participant as in Experiment 1, in the blind field before the eye movement (*bright prime in blind field only* condition), in the sighted field after the eye movement (*dim target in sighted field without blind-field prime* condition), or both before and after the eye movement (*dim target in sighted field with blind-field prime* condition). Both the blind and sighted-field stimuli were presented in the same spatial location. The *bright prime in blind field only* condition repeated Experiment 1's *blind* – *saccade right* condition with the supra-threshold blind-field stimulus presented for 50 ms before the saccade. In the *dim target in sighted field without blind-field prime* condition, the near-threshold sighted-field stimulus was presented 50ms after the beep and remained on the screen for 500 ms, thus ensuring it fell in the sighted visual field after the saccade was completed. For the *dim target in sighted field with blind-field prime* condition, the blind-field stimulus was immediately followed by the sighted-field stimulus. In each condition the stimuli were present on half of the trials. P03 completed fifty blocked trials for each condition. Due to eye movement errors, three trials were excluded from the *bright prime in blind field only* condition, 15 trials were excluded from the *dim target in sighted field without blind-field prime* condition, and no trials were excluded from the *dim target in sighted field with blind-field prime* condition. H3 completed 100 blocked trials in each condition with no exclusions in the *bright prime in blind field only* condition, and 6 trials excluded from both the *dim target in sighted field without blind-field prime* condition and the *dim target in sighted field with blind-field prime* condition.

### 3.2 Results and discussion

**Fig. 4:**
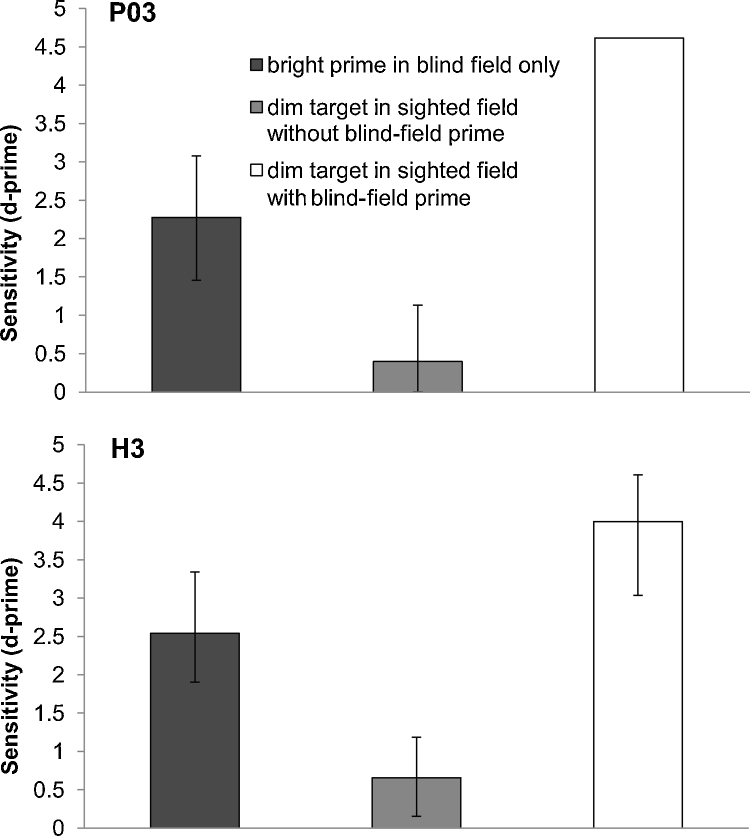
Sensitivity to a high-contrast grating in the blind field shown before saccade (dark grey), and to a low-contrast stimulus shown after the saccade in the sighted field (light grey). When both blind and sighted-field stimuli occur together, sensitivity is greatly elevated (white). Upper panel: P03 results (90% contrast grating the blind field and 1% grating in the sighted field). Lower panel: H3 results (50% contrast grating in the blind field and 2% contrast stimulus in sighted field).

**P03**. Detection was at chance level for the dim sighted-field target alone, but preceding the sighted-field target with the blind-field prime increased sensitivity to the sighted-field stimulus (*dim target in sighted field without blind-field prime*: *d*′ = 0.40, *dim target in sighted field with blind-field prime*: *d*′ = 4.62, *p* < .05). When the blind-field target appeared without a subsequent sighted-field stimulus, detection was similar to the previous experiment (*d*′ = 2.28), see Fig. 4.

**H3**. Detection was at chance level for the dim sighted-field target alone, but preceding the sighted-field target with the blind-field prime increased sensitivity to the sighted-field stimulus (*dim target in sighted field without blind-field prime*: *d*′ = 0.66, *dim target in sighted field with blind-field prime*: *d*′ = 4.0, *p* < .05). When the blind-field target appeared without a subsequent sighted-field stimulus, detection was above chance (*d*′ = 2.54), see Fig. 4. Detection of the blind-field stimulus was higher in this experiment than in Experiment 1, likely due to practice effects.

This experiment illustrated the importance of the integration of pre- and post-saccadic information in trans-saccadic visual processing. In the absence of a prime, a low contrast stimulus in the sighted field could not be reliably detected. But detection was elevated in both participants if the sighted-field stimulus was preceded by a blind-field prime (*dim target in sighted field with blind-field prime* condition). Our interpretation of this boost in performance is that the blind-field stimulus alerted the visual system that a target was present, and when this location was shifted into the sighted field by an eye movement, sensitivity to stimulation here was increased. In this experiment, however, the pre- and post-saccadic stimuli were always presented in the same spatial location. If the observed post-saccadic enhanced sensitivity was specific to the location of the presaccadic stimulus, presenting the presaccadic stimulus in the same location ought to increase sensitivity relative to stimuli in a different location. This hypothesis was tested in the next experiment.

## 4 Experiment 3. Location specificity

In a final experiment we tested the location-specificity of the enhanced sensitivity. Here the supra-threshold blind-field prime (a vertical grating) was followed by the presentation of a near-threshold sighted-field grating tilted 45° to the left or to the right of the vertical meridian. The blind-field prime and sighted-field stimulus were presented in the same or in different locations. Participants were asked to report whether the stimulus was tilted to the left or to the right, and they were informed that the stimulus would appear above or below the second fixation in their sighted visual field, and that it may be difficult to see.

### 4.1 Procedure

**Fig. 5:**
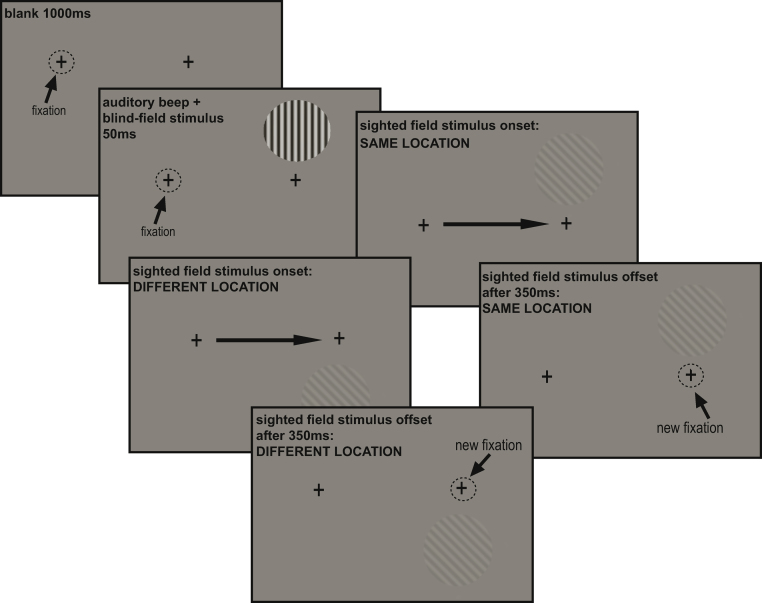
The trial sequence in Experiment 3 for P03. P03 fixates the left crosshair. After 1000 ms, a beep instructs him to shift his eyes to the right crosshair. A 90% grating is shown for 50 ms in one of two locations, above or below the fixation point. The 90% contrast grating is replaced by a 2% grating oriented to the left or right, for 350 ms. It could be in the same or a different location as the 90% grating. The saccade is executed while the 2% grating is still visible.

Participants fixated a cross on the left of the screen for 1000 ms after which an auditory beep sounded which was the cue to saccade to a second cross positioned to the right of the first: P03 (+20°, 0°), H3 (+15°, −6°). Simultaneous with the auditory beep, the pre-saccadic blind-field stimulus was presented in either an upper or lower location for 50 ms. Directly following the blind-field stimulus, the sighted-field stimulus was presented (P03: 350 ms duration at 1% contrast, H3: 500 ms duration at 2% contrast), ensuring it fell within the sighted visual field after the saccade has been completed. The sighted-field stimulus was presented either oriented to the left or the right (45° or 135° from the vertical meridian), and in either an upper or lower location. The upper and lower locations were the same for both the pre-saccadic blind-field prime and the post-saccadic sighted-field target: P03 upper location stimulus near edge – fixation (0°, 9°), lower location stimulus near edge – fixation (0°, −1°), H3 upper and lower locations stimulus near edge – fixation (0°, ±1.5). The blind- and sighted-field stimuli were either shown in the same location (upper or lower) or different locations (one upper, one lower). Both stimuli were present on each trial (see Fig. 5). For H3 we also included a condition in which there was no blind-field stimulus (*no prime* condition) as a baseline measure of her discrimination of the orientation of the sighted-field stimulus. The measure in this experiment was discrimination of orientation, as opposed to simple detection as used in the above two experiments. We therefore report the results from this experiment in terms of percent correct (with chance at 50%) instead of the *d*-prime measure used in the previous two experiments. If the pre-saccadic prime preceded the target in the same location, we predicted that attention would be remapped to that location, thus enabling participants to better acquire visual information, such as orientation, in that location compared to when the prime preceded the target in a different location. For P03 there were 100 trials of the prime and target in the same location and 100 trials of different locations with no excluded trials, and for H3 there were 127 trials in the *no prime* condition after 13 exclusions, 125 trials in the *same location* condition after 15 exclusions, and 128 trials in the *different location* condition after 12 exclusions. As in the above experiments, exclusions were made based on eye movement errors, and statistical inferences were based on bootstrapped confidence intervals.

### 4.2 Results and discussion

**Fig. 6:**
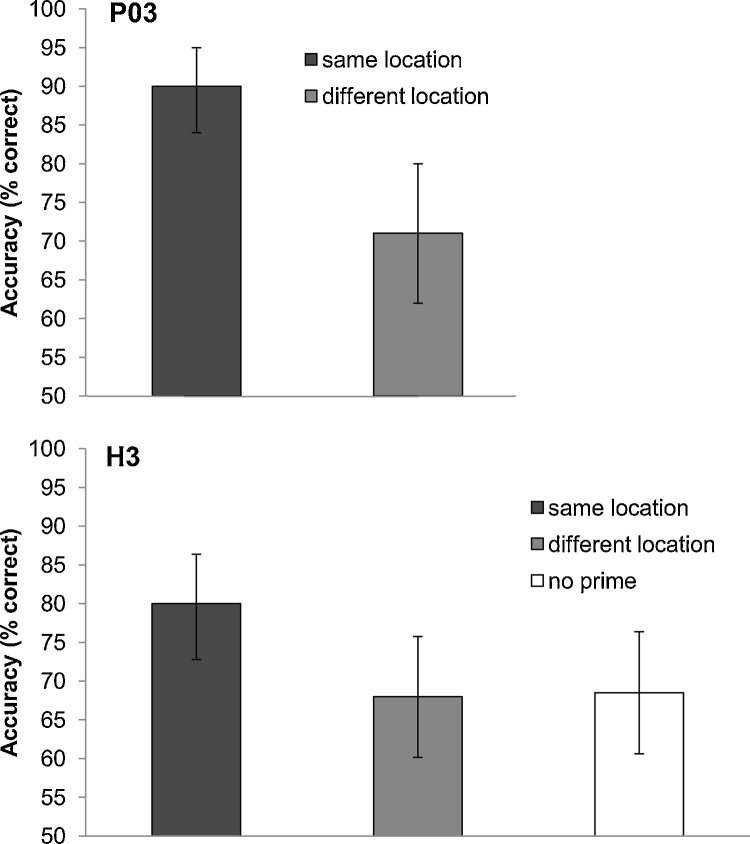
Data for P03 and H3 for Experiment 3. Accuracy to discriminate the orientation of a 2% contrast grating is significantly higher (dark grey) when it appears in the same location as a pre-saccadic blind-field target, relative to a different location (light grey), or when there is no prime presented (white H3 only).

**P03**. Correct discrimination was significantly above chance in both conditions (*same location*: 90%, *different location*: 71%). Orientation discrimination was significantly higher when the pre-saccadic blind-field prime was presented in the same location as the post-saccadic sighted-field target compared to when they were presented in different locations (*p* < .05, see Fig. 6). The duration of the target was brief following the eye movement itself (mean target duration post-saccade = 182 ms), suggesting the benefit for targets appearing in the same location as the pre-saccadic blind-field targets begins immediately following the eye movement.

**H3**. Correct discrimination was significantly above chance in all conditions (*same location*: 80%, *different location*: 68%, *no prime*: 69%). Orientation discrimination was significantly higher when the pre-saccadic blind-field prime was presented in the same location as the sighted-field target compared to when the prime was presented in a different location, or not presented at all (*p* < .05, see Fig. 6).

This experiment established location-specificity of the effect observed in Experiment 2. The presence of the blind-field stimulus appears to have drawn the participants’ attention to a specific location in space. When that location was shifted into the sighted field, subsequent discrimination of stimuli appearing in that location was boosted relative to other locations. The results are consistent with the notion that there is a transfer of target activation just before, or simultaneous with, the eye movement that serves to maintain attention on targets when they shift on the retina.

## 5 General discussion

The results demonstrate that a remapped blind-field prime boosts sensitivity to a near-threshold sighted-field stimulus shown after the saccade. Moreover, accuracy of sighted-field discrimination was significantly higher when a blind-field prime presented before the eye movement was in the same location as the post-saccadic target relative to a different location. Our results suggest that a predictive process supports the continuity of visual information from one fixation to the next, and that these predictions enhance processing at those retinotopic locations in which information is expected to appear. Our interpretation of the results builds on two sets of findings from previous studies: firstly, stimulating frontal eye fields increases visual sensitivity via downward projections to extrastriate cortex (Moore & Armstrong, 2003; Moore & Fallah, 2004); secondly, peaks of activation in LIP and FEF shift to the expected retinotopic locations of targets just before an eye movement (Duhamel et al., 1992; Sommer & Wurtz, 2003). Together these results suggest that an enhancement of processing at the expected retinotopic location of a target should occur just before an eye movement. Our findings are in agreement with recently-demonstrated changes in performance at the expected retinotopic coordinates of attended locations just before an eye movement (Hunt & Cavanagh, 2011; Rolfs et al., 2010).

In Experiment 1, we demonstrated that a stimulus can be remapped from the blind to the sighted field in two hemianopic participants, resulting in increased awareness of that stimulus. Although it is likely that remapping transfers information from the damaged to the intact hemisphere, this remapped information is not akin to actual stimulation in the intact cortex (see Fig. 3 dark (sighted) and dark grey (remapped) bars). With P03, detection was not significantly increased by remapping. On those trials where there was insufficient signal to determine that a stimulus was present, it makes sense that detection would not be affected by remapping. However, on trials where the subject correctly reported target presence, remapping to the sighted field led to a doubling in reports of awareness (from less than 40% to over 80%). With H3, who began from a higher baseline of awareness for the stimulus without an eye movement, the introduction of the saccade led to increased detection and awareness, despite the fact that the stimulus never arrived in the sighted field. The enhanced sensitivity to the stimulus in the *blind* – *saccade right* condition suggests that the represented location of the stimulus shifted from the blind into the sighted field right before the saccade was executed.

In Experiment 2, we demonstrated that the presence of a pre-saccadic blind-field prime facilitates detection of a near-threshold post-saccadic sighted-field target. Indeed, the sighted-field target could not be detected when it was presented without the preceding blind-field prime, and the prime itself was not detected consistently without the presence of the post-saccadic target. The blind-field stimulus was able to alert the visual system to expect a visual target to be present in the sighted field after the eye movement. This information meant that even a weak signal, which was otherwise detected at chance level, could be detected with very high accuracy. Consistent with this interpretation, the final experiment demonstrated that when both the pre- and post-saccadic targets were presented in the same spatial location, the orientation of the sighted-field target was correctly discriminated significantly more often than when the stimuli appeared in different locations. This shows that the remapping process leads not simply to the summation of signals from before and after the eye movement, but to a spatially-specific shift of attention to the expected location of the post-saccadic stimulus, boosting visual sensitivity at that location.

One possible alternative explanation for the results of Experiment 1 is the well-known shift of visual attention to saccade targets that immediately precedes an eye movement (e.g. Remington & Pierce, 1984; Rizzolatti, Riggio, Dascola & Umiltá, 1987). That is, shifting attention to the saccade target alone might have boosted sensitivity to stimuli in the vicinity of the saccade target, which may serve to increase awareness of the blind-field stimulus. Nevertheless, a shift of attention to the saccade target cannot account for the results of Experiments 2 and 3, in which there was enhancement of processing at the post-saccadic sighted-field location. If attention shifted out into the blind field to the saccade target before the saccade, there is no reason to expect this shift alone to enhance sensitivity in the sighted field only a few moments later. This is not to say that a pre-saccadic shift of attention did not occur, only that it would have to be followed by a shift back to the fovea immediately before the saccade for this mechanism to effectively boost performance at the fixation. However, even if this shift back to the fovea did occur, it could not account for the fact that performance was only enhanced when there was a blind-field stimulus present in Experiment 2, and that the enhancement was specific to the location of that stimulus in Experiment 3.

Previous research has shown that a bright distractor in the temporal region of the blind hemifield slows saccadic responses to a target in the sighted hemifield (Rafal et al., 1992). The findings provided evidence for the role of ipsilesional retinocollicular pathway in saccade initiation (but see Williams, Azzopardi & Cowey, 1995). Furthermore, the retinocollicular pathway has been implicated in tasks involving spatial summation in cases of hemispherectomy. Two of four hemispherectomised patients studied showed faster responses to a stimulus in the sighted visual field when it was accompanied by a second task-irrelevant target in the blind field (Tomaiuolo, Ptito, Marzi, Paus & Ptito, 1997). More recent functional imaging findings have shown the existence of strong collicular projections to the contralesional hemisphere, only in those hemispherectomised patients who have shown a significant redundant target effect (Leh, Mullen & Ptito, 2006). Although a collicular orienting response could explain the above-chance detection of stimuli in the blind field in our participants, it is not sufficient to explain our main finding, which is the enhancement of post-saccadic sensitivity to targets in the sighted field following the blind-field prime. It is important to keep in mind that the targets to be detected/discriminated in our Experiments 2 and 3 were presented at different retinal locations than the blind-field primes that preceded them. Therefore, the findings in experiments 2 and 3 are consistent with not only an orienting response towards stimuli presented in the blind field, but also a *re-orienting* back into the sighted field before the saccade, leading to better discrimination of targets appearing in the same spatial location as blind-field primes.

Our results show remapping to be intact for stimuli in the blind field of the two patients tested. This confirms previous results suggesting remapping in early visual areas to be minimal (e.g. Nakamura & Colby, 2002). It is also consistent with research demonstrating that visual stability relies, at least in part, on a pathway between the superior colliculus and the frontal cortex (Sommer & Wurtz, 2008). The superior colliculus has efferent projections to the oculomotor nuclei to initiate eye movements, and it also projects to the FEF via the thalamus, and this colliculo-frontal pathway has been shown to carry a corollary discharge signal, that is, a copy of the efferent motor command that is used for remapping (Sommer & Wurtz, 2002, 2006). These eye movement generation and efference copy pathways are intact in our patients, and support remapping of stimuli out of the damaged hemifield. In contrast, the importance of the right parietal lobe in remapping has been demonstrated in studies on patients with parietal lesions. In a task involving the remapping of inhibitory tags in visual search (inhibition of return, IOR) neurologically intact participants showed IOR in both retinotopic and spatiotopic locations, demonstrating that the tag had been remapped in spatial coordinates. Right parietal lesioned patients, however, showed IOR only in retinotopic coordinates, indicating the involvement of the intraparietal sulcus in remapping (Sapir, Hayes, Henik, Danziger, & Rafal, 2004). Remapping has been demonstrated in extrastriate visual areas, but to a lesser extent than in FEF and LIP. Using fMRI the percentage of voxels showing a remapping response seems to decrease with decreasing connectivity to LIP, with 61% in V3A, 23% in V2, and only 17% in V1 (Merriam et al., 2007). With direct recordings from visual cells, just 2% of neurons in V1 showed a remapping response (Nakamura & Colby, 2002). Therefore it has been proposed that these early visual areas do not play a major role in remapping. Our results support this conclusion to the extent that brain areas that support conscious vision corresponding to our patients’ blind fields do not seem to be necessary for the signal to be remapped, given that we found evidence of remapping of stimuli presented in the blind field. However, this does not mean that early visual areas play no role in trans-saccadic visual perception. The remapping process generates an expectation that a target stimulus is about to appear in a specific location, and input to V1 that is consistent with that expectation is what bridges perception from one fixation to the next to form a continuous, uninterrupted visual experience.
